# Structure-Function Correlative Microscopy of Peritubular and Intertubular Dentine

**DOI:** 10.3390/ma11091493

**Published:** 2018-08-21

**Authors:** Tan Sui, Jiří Dluhoš, Tao Li, Kaiyang Zeng, Adrian Cernescu, Gabriel Landini, Alexander M. Korsunsky

**Affiliations:** 1 Department of Mechanical Engineering Sciences, University of Surrey, Guildford GU2 7XH, UK; 2 TESCAN Brno, s.r.o., Libušina třída 1, 623 00 Brno, Czech Republic; jiri.dluhos@tescan.cz; 3 Department of Mechanical Engineering, National University of Singapore, Singapore 117576, Singapore; peach.fish.b@gmail.com (T.L.); mpezk@nus.edu.sg (K.Z.); 4 Neaspec GmbH, Bunsenstr. 5, Martinsried, D-82152 Munich, Germany; adrian.cernescu@neaspec.com; 5 School of Dentistry, College of Medical and Dental Sciences, University of Birmingham, 5 Mill Pool Way, Edgbaston, Birmingham B5 7EG, UK; G.Landini@bham.ac.uk; 6 Department of Engineering Science, University of Oxford, Parks Road, Oxford OX1 3PJ, UK

**Keywords:** peritubular dentine (PTD), intertubular dentine (ITD), FIB-SEM-EDS tomography, tapping mode AFM, s-SNOM, parabolic stiffness-volume fraction correlation function

## Abstract

Peritubular dentine (PTD) and intertubular dentine (ITD) were investigated by 3D correlative Focused Ion Beam (FIB)-Scanning Electron Microscopy (SEM)-Energy Dispersive Spectroscopy (EDS) tomography, tapping mode Atomic Force Microscopy (AFM) and scattering-type Scanning Near-Field Optical Microscopy (s-SNOM) mapping. The brighter appearance of PTD in 3D SEM-Backscattered-Electron (BSE) imaging mode and the corresponding higher grey value indicate a greater mineral concentration in PTD (~160) compared to ITD (~152). However, the 3D FIB-SEM-EDS reconstruction and high resolution, quantitative 2D map of the Ca/P ratio (~1.8) fail to distinguish between PTD and ITD. This has been further confirmed using nanoscale 2D AFM map, which clearly visualised biopolymers and hydroxyapatite (HAp) crystallites with larger mean crystallite size in ITD (32 ± 8 nm) than that in PTD (22 ± 3 nm). Correlative microscopy reveals that the principal difference between PTD and ITD arises primarily from the nanoscale packing density of the crystallites bonded together by thin biopolymer, with moderate contribution from the chemical composition difference. The structural difference results in the mechanical properties variation that is described by the parabolic stiffness-volume fraction correlation function introduced here. The obtained results benefit a microstructure-based mechano-chemical model to simulate the chemical etching process that can occur in human dental caries and some of its treatments.

## 1. Introduction

Human dentine is a tough natural mineralized material which consists of ~50% by volume of mineral hydroxyapatite (HAp), ~30% organic matter, and up to ~20% water. It has a typical well-oriented microstructure consisting of an arrangement of dentinal tubules filled with odontoblast processes or their remnants with an area density of around (19–45) × 1000/mm^2^ and a mean diameter range of 0.8–2.5 μm. Tubules extend throughout the entire dentine thickness, from the dentine-enamel junction (DEJ) to the pulp [1,2,3]. The tubules are surrounded by two distinct dentinal phases: peritubular dentine (PTD) and intertubular dentine (ITD). The greater part of the dentine volume is occupied by ITD which is a composite consisting of collagen fibrils discontinuously reinforced with nanoplatelets of carbonated HAp. This arrangement makes the dentine tough and strong. The PTD is more mineralized than ITD, contains no collagen, and is harder and stiffer than ITD [4,5,6].

Considerable efforts have been directed to achieve a detailed observation of the dentine tubules features across the scales from the (sub) millimetre to micro-scale, and further to nanoscale, using the still-developing advanced characterization tools and cutting-edge nanometre resolution microscopy techniques, such as synchrotron X-ray beams and focused electron and ion [7,8,9,10,11,12]. However, there is still a gap of understanding the difference between ITD and PTD in a correlative way, as an integral of both structural and compositional difference in 3D in micron scale and their link with the underlining structure of HAp crystallites at finer scale. Focal spot sizes of a few hundred nanometres synchrotron X-ray beam have been used to map 2D elemental distribution and scattering from HAp using X-ray diffraction and spectroscopy techniques on lamella samples with thickness ~1 μm from bovine dentine [7,8]. X-ray diffraction-based tomography has been used to visualise the spatial distribution of nanocrystalline HAp around the dentinal tubules, but the technique has not been able to reveal a contrast between ITD and PTD [9]. Ptychographic X-ray imaging tomography has been applied to distinguish and quantify the differences in mineralization density between ITD and PTD, but no direct link has been made with the elemental distribution and the underlying nanocrystalline HAp structure [10]. Focused Ion Beam (FIB)-Scanning Electron Microscopy (SEM) tomography has been recently reported to visualise 3D dentinal tubules, but so far it has failed to capture and reconstruct the contrast between ITD and PTD [11,12]. Other techniques that can be used to enrich the feature of dentinal tubules include Energy Dispersive Spectroscopy (EDS) and Time-of-flight Secondary Ion Mass Spectroscopy (TOF-SIMS) which specifies the spatial elemental distribution [13,14], and tapping mode Atomic Force Microscopy (AFM), which has demonstrated to deliver nanometre resolution characterization of organic-inorganic two-phase in biomaterials [15] and has been widely used to image the structure and mechanical properties of dentinal tubules [16,17,18,19,20]. Nevertheless, to our best knowledge, no reports have been found regarding the direct size quantification of nanocrystalline HAp both in PTD and ITD by tapping mode AFM, and the correlation with 3D information on the spatial distribution, morphology and elemental distribution in ITD and PTD by simultaneous FIB-SEM milling and Energy Dispersive Spectroscopy (EDS) for tomography.

On moving from the DEJ towards the pulp in the human tooth, the diameter of the dentinal tubules is known to increase, whilst the spacing of the dentinal tubules decreases. This mainly results in the reduction of the volume fraction ratio of the ITD with respect to the PTD (and overall). Nevertheless, it is also clear that there is a persistent and consistent variation of the *local* structural, chemical and mechanical properties from the central tubule hollow through the PTD and into the ITD. Our present study is aimed at revealing the origins of the difference between the ITD and PTD, with a clear focus on the fine scale characterization and analysis of a representative single dentinal tubule. We explore the principal difference between ITD and PTD using simultaneous FIB-SEM-EDS nanotomography and AFM/s-SNOM as a correlative technique to achieve 3D spatially resolved structural and compositional characterization of the arrangement of mineral and organic phases within ITD and PTD of human dentine from micron to nanometre scales. A parabolic stiffness-volume fraction correlation function is proposed to link the structural features with mechanical properties.

## 2. Materials and Methods

### 2.1. Sample Preparation

A freshly extracted human third molar with no dental restorations, caries or damage was used for this study (ethical approval obtained from the National Research Ethics Committee; NHS-REC reference 14/EM/112, Consortium reference BCHCDent 332.1531.TB). A low-speed diamond saw (Isomet Buehler Ltd., Lake Bluff, IL, USA) was used to cut the sample into 1 mm thick cross-sectional slices in the bucco-lingual orientation incorporating dentine and enamel, and polished by a series of polishing papers to obtain the final sample. To eliminate the charging effects on the non-conducting dental slice sample during the electron image acquisition, the sample was coated with 10 nm thin film of Au in vacuum using a mini sputter coater (SC7620, Quorum Technologies, Laughton, East Sussex, UK).

### 2.2. 3D FIB-SEM-EDS Tomography

A “U-shaped trench” was first prepared using the FIB-SEM instrument TESCAN LYRA 3 GMU (Brno, Czech Republic) to expose the volume of interest (VOI), and a layer of platinum was deposited on the VOI to protect the sample and also to reduce the charging effects. The serial sectioning experiment was performed on the volume of interest (VOI) (~20 × 20 × 10 µm^3^) by removing 200 nm thick layers of material by FIB milling at 30 keV and 505 pA of current, followed by optimised in-Beam BSE imaging at 10 kV on each newly exposed surface (imaging pixel size of 15 nm in both *x*- and *y*-directions) with simultaneous EDS mapping. As a result, a series of 36 consecutive BSE images with good contrast (61.4%) and brightness (84.9%), and EDS images for each element were obtained, providing input for 3D reconstruction with dimensions: *x* (16.9 µm), *y* (21.6 µm), and *z* (7.2 µm). The reconstruction and visualisation were achieved in the *3D Tomography* module provided as part of the Tescan FIB-SEM microscope software package (2016).

In addition to the above mapping, 2D analytical SEM-EDS was performed on the front surface over a smaller 15 × 15 µm^2^ region of interest (ROI) of FIB milled trench using Oxford Instruments (High Wycombe, UK) X-Max 150 EDS detection system. The elemental distribution maps of Ca, O, P, C, and Mg were obtained from the ROI with the pixel resolution of 55 nm in both *x*- and *y*-directions, allowing the weight percentage of individual elements to be determined from the analysis of the EDS spectra.

### 2.3. 2D Tapping Mode AFM

Tapping mode AFM induces small range probe tip oscillation that changes in amplitude and phase not only depending on the sample surface topography, but also on the properties and the mode of interaction with the sample material. High resolution topography (height image), amplitude image, and phase image were obtained using a commercial AFM instrument (MFP-3 D, Asylum Research, Oxford Instruments, High Wycombe, UK). Amplitude image reveals morphological details and phase image is used to identity regions with different material phases (composition, elasticity or others). A large area (10 × 10 µm^2^) was mapped initially to identify PTD and ITD regions at the sample surface. This was followed by detailed height and phase mapping (3 × 3 nm^2^ pixel size) both for PTD and ITD regions, with the scan areas of 700 × 700 nm^2^.

### 2.4. s-SNOM Imaging

Scattering-type Scanning Near-Field Optical Microscopy (s-SNOM) employs the combination tapping mode AFM with laser focusing at the tip followed by pseudoheterodyne analysis to beat the diffraction limit by 1000 times, achieving wavelength-independent 10 nm resolution combined with selective sensitivity to molecular species by tuning the excitation to selected absorption bands. In the current study, data collection was carried out at neaspec GbmH (Munich, Germany) using 1020 cm^−1^ illumination to excite the ν3 phosphate absorption band in HAp.

## 3. Results

### 3.1. Structural Difference between PTD and ITD

**Figure 1:**
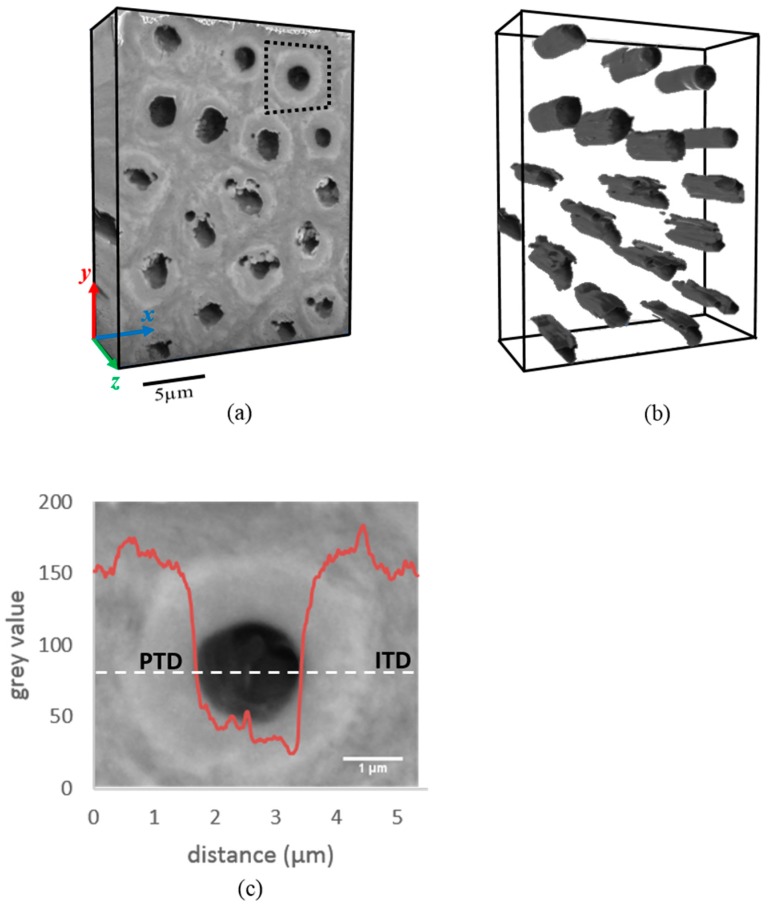
3D Focused Ion Beam (FIB)-Scanning Electron Microscopy (SEM) tomography of a representative volume of dentine. (**a**) 3D reconstruction of the dentine structure composed of tubules, peritubular dentine (PTD) and intertubular dentine (ITD), distinguishable by the contrast in greyscale intensity. (**b**) 3D spatial distribution of dentinal tubules (dark grey). The morphology of a typical tubule is approximately cylindrical with ~1.6 µm diameter section. Tubules are seen to run parallel to each other with ~5 µm inter-tubule spacing. (**c**) The variation of grey scale values across the centre of a representative single dentinal tubule (marked by the dashed region in Figure 1a). Note the gradient in the grey value observed in the boundary between PTD and ITD. The thickness of PTD is ~1 µm, comparable to the diameter of the tubule.

The 3D rendering of the spatial distribution of PTD and ITD is displayed in Figure 1a by reconstructing a sequence of BSE images obtained by sequential FIB millings. The overall reconstructed volume is 16.92 × 21.6 × 7.2 µm^3^ and dentinal tubules are also displayed in Figure 1b. The tubules are seen to have an overall shape of approximately circular cylinders that run parallel with each other, having a mean cross-sectional diameter of ~1.6 µm and mean spacing of ~5 µm. The features associated with a single tubule (highlighted by the black dashed square in Figure 1a) were further explored in detail to reveal the variation of the grey value (intensity) across PTD, ITD, and within the tubules. The mean grey value varies from the value ~152 in the ITD to ~160 in the PTD, and drops down to ~44 upon reaching the hollow tubules (see Figure 1c).

In passing, we note that the underlying data from FIB-SEM is three-dimensional, i.e., provides 3D structural and chemical information, and contains both longitudinal section (*y-z* plane) and cross-sectional (*x-y* plane) information. For the purposes of studying the radial variation as discussed above, i.e., for radial lines taken from either transverse or longitudinal sections, the same underlying data is used, confirming that the conclusions drawn in this way are reliable and robust.

**Figure 2:**
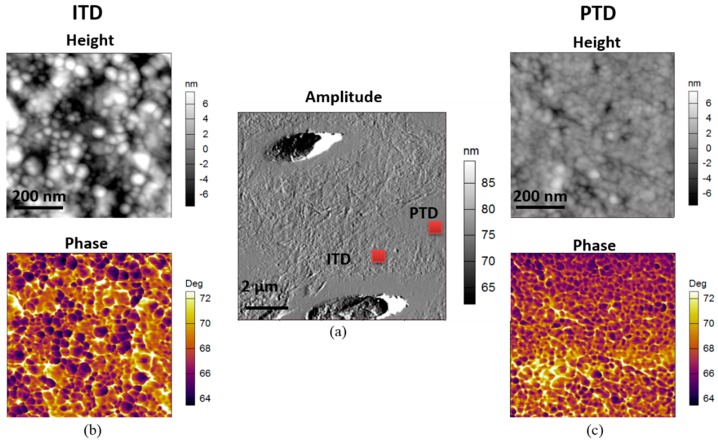
Illustration of the morphology of nanocrystalline hydroxyapatite (HAp) using 2D tapping mode Atomic Force Microscopy (AFM) mapping of dentine. (**a**) Amplitude image (middle) of a 10 × 10 µm^2^ region containing tubules, PTD and ITD. The height and phase images of a 700 × 700 nm^2^ region of interest (ROI), marked by the red squares in (**a**) of ITD and PTD are presented respectively in columns (**b**,**c**) above. Both the height and phase images reveal significantly larger nanocrystalline HAp size in ITD than in PTD. The phase image additionally reveals that the nanocrystalline HAp (dark regions) are bonded by biopolymer phases (bright regions) lying at the boundaries of the mineral grains.

The conventional AFM image of a typical 10 × 10 µm^2^ region containing tubules shown in Figure 2a reveals the difference between PTD and ITD in terms of surface topography (height). Small scale tapping mode AFM was used on the regions marked with red squares (PTD and ITD) to extract the height and phase images, as presented in Figure 2b,c respectively. It is clear from the comparison that both images reveal significantly larger nanocrystalline HAp size in ITD than in PTD with the mean and range (quantified using ImageJ (version 1.50e), National Institutes of Health, Bethesda, MD, USA) particle analysis tool as 32 ± 8 nm and 22 ± 3 nm in the ITD and PTD, respectively. The fine scale features in the phase image additionally reveal the nanocrystalline HAp grain morphology (dark region), and particularly the organic phase biopolymer or biophase boundaries (bright region) surrounding HAp nanocrystallites.

**Figure 3:**
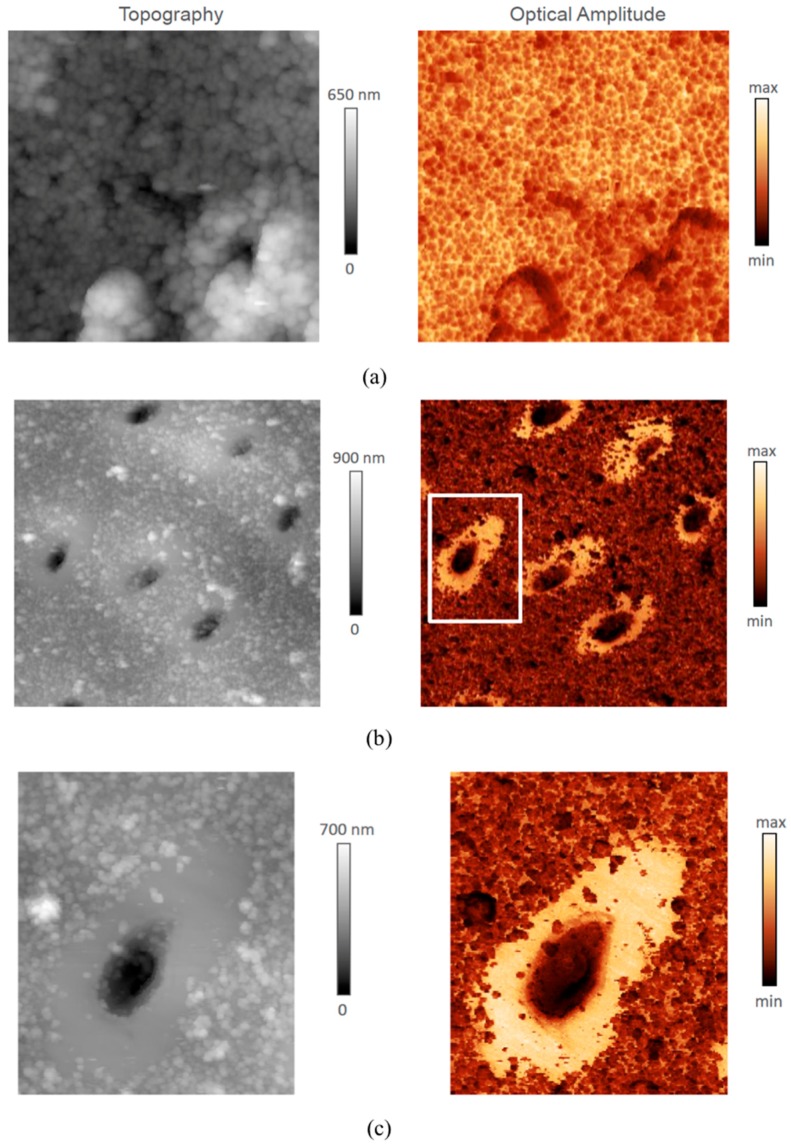
Dentine mapping using scattering-type Scanning Near-Field Optical Microscopy (s-SNOM) with 1020 cm^−1^ excitation of the ν3 phosphate absorption band in hydroxyapatite (HAp). (**a**) Topography (left column, nm) and Optical Amplitude (right column, arbitrary units of intensity) images of a 5 × 5 µm^2^ region of ITD revealing the grain structure. (**b**) A large area (20 × 20 µm^2^) scan of a region containing multiple dentinal tubules. The preparation of the sample for microscopic investigation involve cutting and polishing. Despite best care taken, some detachment and re-adhesion of HAp particles to the surface is likely. This is primarily visible in the ITD which contains a higher volume fraction of organic material to which these particles get attached. (**c**) Zoomed in image (6 × 7 µm^2^, indicated in (**b**)) of the area around an individual tubule emerging at the surface obliquely to reveal an “avocado-shaped” region of PTD. Note the fine-grained appearance of PTD structure.

In Figure 3a–c, s-SNOM mapping results are shown in two columns representing topography (height) and optical amplitude, respectively. Figure 3a shows the images of a 5 × 5 µm^2^ region of ITD. The grain structure is revealed particularly clearly in the optical amplitude image, illustrating the potential of this approach for the study of human dental tissues and bone. Figure 3b shows a large area (20 × 20 µm^2^) scan of a region containing multiple dentinal tubules. The particles present at the surface of ITD may be related to sample preparation artefact, and reveal the larger volume fraction of “organic glue” in ITD compared with PTD. Figure 3c shows a zoomed in image (6 × 7 µm^2^, indicated in (b)) of the area around an individual tubule that emerges at the surface obliquely. An “avocado-shaped” region of PTD is particularly clearly revealed in the optical amplitude image. The smoother surface appearance of PTD in Figure 3c is indicative of the very fine-grained structure of this region.

### 3.2. Chemical Composition Difference between PTD and ITD

**Figure 4:**
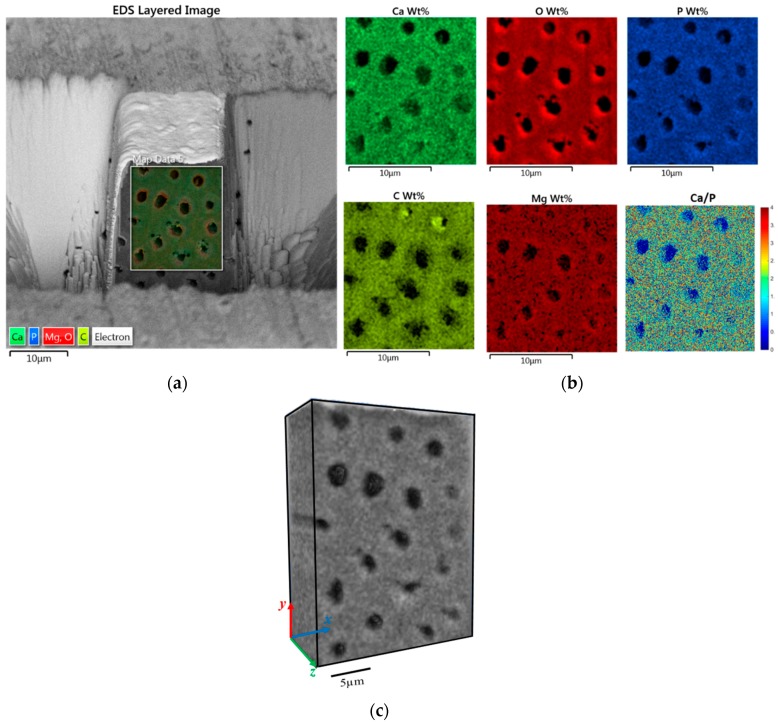
2D-3D FIB-SEM-Energy Dispersive Spectroscopy (EDS) technique reveals the spatial distribution of chemical elements in dentine. (**a**) EDS layered image on the front surface of FIB milled trench: the dark regions correspond to the dentinal tubules. Weight percentage (concentration) maps of different chemical elements (Figure 3b) are superimposed on the front surface of dentine. PTD and ITD can be distinguished by the contrast in colour intensity, indicating the difference in elemental population in the two regions. (**b**) EDS weight percentage maps for individual elements Ca, O, P, C, and Mg surrounding PTD and ITD. C dominates in the ITD while other elements dominate in the PTD. The image on the bottom right is a quantitative map of the Ca/P ratio. (**c**) Visualisation of the 3D distribution of element P in dentine. The somewhat lower resolution compared to BSE imaging is due to the larger volume for detection.

Figure 4a shows a 2D back scattered electron image superimposed with 2D SEM-EDS elemental mapping on a cross section of the sample. PTD and ITD are clearly distinguished by the contrast in colour intensity that corresponds to elemental concentration. The detailed elemental spatial distribution maps for major elements Ca, O, P, C and Mg are shown in Figure 4b. There is clear evidence of enrichment in elements O and Mg and moderate enrichment in the elements Ca and P in the PTD, which is in agreement with previously reported observations [21] as associated with nanocrystalline HAp, whereas C is more concentrated within ITD, indicating the greater presence of organic components in this region. To quantify the mineralization content in the PTD and ITD, the Ca/P ratio was calculated from the raw weight percentage maps and plotted in Figure 4b (bottom right), revealing the mean value ~1.8 for this parameter both in the PTD and ITD, which is in good agreement with the literature [22], but also indicates that PTD and ITD cannot be distinguished solely from the chemical concentration.

Making use of the 2D SEM-EDS approach of dentinal tubules, we explored further their 3D spatially resolved elemental concentration, which was made from the set of 2D EDS maps simultaneously acquired while FIB-SEM tomography was performed. Figure 4c shows the 3D spatial distribution of a representative element P. Note that the point resolution for each slice in the 3D EDS map is lower than that in the 2D configuration map (Figure 4a), because of the different sample-detector configuration that had to be employed for tomographic FIB slicing, and the need to optimize the milling rate and acquisition parameters for 3D data collection. The inhomogeneity of the density (in grey scale values) can be perceived in the *x-y* plane in Figure 4c. PTD appears slightly lighter than ITD, indicating more concentration of mineral element P in PTD. This is similar to the findings in 2D map, but with enriched feature through the thickness direction, although no significant variation of mineral element P can be observed. Similar results are observed for other elements are thus not shown here.

## 4. Discussion

A large number of advanced microscopy techniques have been applied to observe the ITD and PTD, ranging from Transmission Electron Microscopy (TEM) to advanced X-ray imaging. Nevertheless, the insights obtained into the architecture of dentinal tubules obtained from these observations remain somewhat inconclusive. For TEM, sample preparation is known to cause tissue modification. In scanning transmission X-ray microscopy (STXM), the sample thickness is likely to exceed the beam spot size by a factor of ×5 or more, so that integration through the sample thickness reduces the effective resolution of the ultrastructure variation from ITD and PTD. In addition, X-ray reciprocal space mapping and reconstruction provide only indirect structural and morphological information of HAp nanocrystals, and therefore fail to describe the micro-architecture as far as the organic biopolymer matrix and HAp reinforcements are concerned.

**Figure 5:**
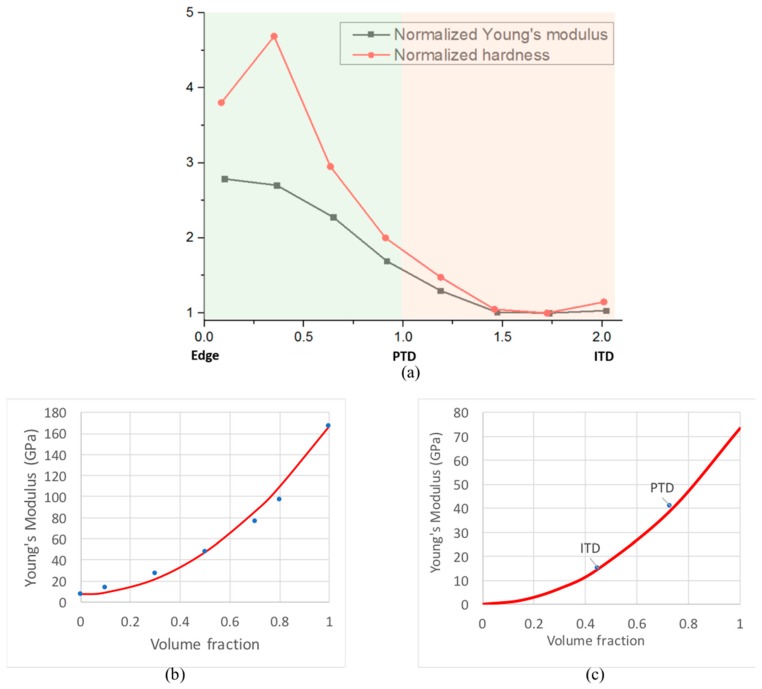
Young’s modulus and hardness of PTD and ITD. (**a**) The values from Ziskind et al. [22] in *y*-axis were normalized with respect to the ITD values. The horizontal axis represents normalized position, so that 0 and 1 on the *x*-axis correspond to the edge of the tubule and the boundary of PTD with ITD, respectively. The region of PTD on the left and ITD on the right are highlighted in different backgrounds. (**b**) Young’s modulus of a particle-reinforced composite calculated using FEM (Finite Element Method) by Fritzen and Boehlke [20] that reveals the steep (faster than the Voigt linear rule-of-mixtures) rise in stiffness with volume fraction. The parabolic fit (in red line) to the data points (in blue dots) shown by the curve is introduced in the present study, and discussed in the text. (**c**) The parabolic description of the stiffness-volume fraction dependence applied to ITD and PTD values shown by the markers.

The brighter appearance of PTD in 3D SEM-BSE imaging mode and the corresponding higher grey value indicate a higher degree of mineral concentration in PTD compared to ITD in human dentine [23]. However, the 3D elemental distribution and high resolution, quantitative 2D elemental map of the Ca/P ratio analysis fail to reveal distinct contrast between PTD and ITD, suggesting that the principal difference between these two regions concerns structural, rather than compositional parameters: the mean grain size, packing density, accompanied by some change in the volume fraction of the mineral phase. This has been further qualitatively confirmed using nanoscale 2D AFM map, which provides a clear visualisation of biopolymers and mineral phases and a direct observation that mean crystallite size in ITD is larger than that in PTD, which is consistent with the studies using XRD and TEM [24,25]. Conclusion is drawn from the above systematic observations that the difference between ITD and PTD is primarily from the nanoscopic packing density of HAp crystallites with moderate contribution from the chemical composition difference. The insights obtained and reported in this study provide a possible means of explaining an apparent ‘paradox’ [26,27], that PTD is dissolved faster than ITD under the attack of carious dental plaque acidic medium. The finer PTD particles with the higher surface-to-volume ratio dissolve more rapidly when HAp crystallites become surrounded by acid medium. From the mechanical perspective, the finer-grained, more highly mineralized structure of PTD provides a firm basis for explaining the higher Young’s modulus (~40 GPa in the PTD and ~17 GPa in the ITD) and mean hardness (1.34 ± 0.5 GPa in the PTD and 0.60 ± 0.2 GPa in the ITD) of PTD compared to ITD that has been reported previously [22]. The variation of Young’s modulus and hardness from the edge of the tubules to PTD and ITD is plotted in Figure 5a.

The correlation between the degree of mineralization (HAp volume fraction) and tissue (composite) stiffness is an important relationship that needs to be quantified. However, the classical approaches to composite stiffness evaluation using the Voigt, Reuss, Hill and Hashin–Shtrikman bounds fail to describe this correlation adequately. The study by Fritzen and Boehlke [28] present the results of a careful FE analysis of the stiffness of a particle-reinforced metal matrix composite. The structure consists of approximately equiaxed reinforcing particles bonded with softer matrix. The authors developed the procedures for generating FE meshes to represent the microstructure corresponding to different volume ratios of reinforcement, as well as the algorithms for evaluating composite stiffness parameters, such as bulk modulus, shear modulus, and the coefficient of thermal expansion. Their results for the stiffness shown in Figure 16b of paper [28] are re-plotted in Figure 5b here in blue dots, together with the simple parabolic fit in red line given by

(1) $$E_{C}=E_{M}+\left(E_{P}-E_{M}\right)f^{2}$$

where $E_{C}$ denotes the composite stiffness, $E_{M}$ that of the matrix and $E_{P}$ that of the particles, and *f* is the volume fraction of the latter. Unlike the very wide Voigt–Reuss bounds, and even the tighter Hashin–Shtrikman bounds, this approximation does not contain any free parameters, yet matches the results very closely. The remarkable quality of the fit, and the clear physical meaning of the terms appearing in the above formula provide the encouragement to apply this approach to the consideration of Young’s modulus variation between ITD and PTD.

Figure 5c shows the result of applying the parabolic stiffness-volume fraction correlation (Equation (1)) to dentine. The value of stiffness of the organic matrix was taken to be 0.3 GPa, approximately equal to that of the collage Type I fibers [29], whilst the stiffness of the reinforcement was taken to be equal to 75 GPa, corresponding to that of highly mineralized enamel [30]. The mineral volume fraction is reported to be 0.45 in PTD and 0.73 in ITD [10]. The quality of agreement suggests that this formula can be used not only to estimate the local stiffness of dentinal tissue as a function of HAp volume fraction, but also to perform inverse analysis, i.e. evaluate the local volume fraction from spatially resolved stiffness measurement, e.g. by nanoindentation.

Returning to Figure 5a, it is worth noting that the variation of hardness shows a higher ratio (>4) between PTD and ITD than the Young’s modulus (<3). This can be explained by observing that the increased mineral content and grain refinement act together to increase the hardness by virtue of the volume fraction increase (that can be captured in more detail using Eshelby-based hierarchical modelling [31]), and the Hall–Petch grain refinement effect [32].

Simultaneous FIB-SEM-EDS structural and elemental tomography allowed 3D reconstruction of nanoscale images of individual tubules on the basis of the grey value contrast from in-beam BSE detector and elemental concentration from EDS detector, including the distinction between PTD and ITD. This made it possible to establish the spatially resolved association between the tubule structure, mineralization density, and elemental distributions. A broader application of the proposed approach in this study can be the complex dynamic chemical processes such as de- and re-mineralization (dissolution and formation of different crystallite phase) that can occur in human dental caries and some of its treatments.

## 5. Conclusions

In summary, correlative FIB-SEM-EDS nanotomography technique enabled us to characterize both 2D and 3D spatially resolved structural and compositional information around dentinal tubules, in particular within ITD and PTD, which provided the basis for understanding Nature’s hierarchical design of human dentine regarding grain size and spread, mineral content volume fraction, biointerphase boundary thickness as well as Ca/P ratio. Principal difference between ITD and PTD is primarily from the structural variation of HAp crystallites at nanoscale rather than the local compositional variation. The parabolic stiffness-volume fraction correlation function enables to link between the structural features and mechanical properties, which opens up the establishment of a microstructure-based mechano-chemical model to qualitatively simulate the chemical etching process by altering the structure and its influence on the mechanical response of PTD and ITD.
